# Chronic Low Quality Sleep Impairs Postural Control in Healthy Adults

**DOI:** 10.1371/journal.pone.0163310

**Published:** 2016-10-12

**Authors:** Fabianne Furtado, Bruno da Silva B. Gonçalves, Isabela Lopes Laguardia Abranches, Ana Flávia Abrantes, Arturo Forner-Cordero

**Affiliations:** 1 Department of Mechatronics, Escola Politécnica, University of Sao Paulo, São Paulo, Brazil; 2 Federal Institute of Education, Science, and Technology of Southeast of Minas Gerais, Barbacena, Minas Gerais, Brazil; 3 Department of Psychiatry, Universidade Federal de Sao Paulo (Unifesp), Sao Paulo, Brazil; Associazione OASI Maria SS, ITALY

## Abstract

The lack of sleep, both in quality and quantity, is an increasing problem in modern society, often related to workload and stress. A number of studies have addressed the effects of acute (total) sleep deprivation on postural control. However, up to date, the effects of chronic sleep deficits, either in quantity or quality, have not been analyzed. Thirty healthy adults participated in the study that consisted of registering activity with a wrist actigraph for more than a week before performing a series of postural control tests. Sleep and circadian rhythm variables were correlated and the sum of activity of the least active 5-h period, L5, a rhythm variable, obtained the greater coefficient value with sleep quality variables (wake after sleep onset WASO and efficiency sleep). Cluster analysis was performed to classify subjects into two groups based on L5 (low and high). The balance tests scores used to asses postural control were measured using Biodex Balance System and were compared between the two groups with different sleep quality. The postural tests were divided into dynamic (platform tilt with eyes open, closed and cursor) and static (clinical test of sensory integration). The results showed that during the tests with eyes closed, the group with worse sleep quality had also worse postural control performance. Lack of vision impairs postural balance more deeply in subjects with chronic sleep inefficiency. Chronic poor sleep quality impairs postural control similarly to total sleep deprivation.

## Introduction

Sleep is essential for health and sleep disturbances (insufficient duration, poor quality, and irregular timing) can cause a number of disorders [1]. With respect to sleep research, the duration is the most frequently investigated measure because it is easily obtained by an individual inquiry. The appropriate sleep duration for adults has been suggested to be more than 9 hours per night [2], while other authors claimed that a minimum of 7–8 hours of sleep would be sufficient [3]. Several factors, such as work demands, can reduce the sleeping duration below the recommended values eliciting a wide range of effects on mood, cognitive and motor functions [4].

Sleep disorders have a negative impact on postural control (PC) and the standing posture is crucial to perform different tasks, from reaching to locomotion [5]. Moreover, sleep disorders and subsequent PC deterioration are related to work accidents, like driving accidents [6] and falls among frail populations, such as the elderly [7].

Several studies reported that PC deteriorated after one night without sleep [8–16]. However, other studies only found significant differences in PC with sleep deprivation (SD) when the eyes were closed [17–23]. It is well-known that PC performance decreased with eyes closed [24] and this effect might be accentuated with SD [17–23].

Nevertheless, most of the evidence showing that PC was impaired by sleep deprivation was obtained from experiments with participants that were prevented from sleeping for one complete night, that is an acute SD. Furthermore, most of the studies did not monitor objectively the sleep conditions of the participants in the days prior to the tests.

Partial SD or sleep restriction during several days or weeks is a relatively common condition that consists of sleeping below the basal needs and it can occur in three ways [3]. In the first one, sleep fragmentation, sleep is not physiologically consolidated and the normal sequence of sleep stages is disrupted, resulting in less time in consolidated physiological sleep, because fragmentation by brief arousals decreases sleep quality. The second type involves loss of specific physiological sleep stages, and it is referred to as selective sleep stage deprivation. It can occur if sleep fragmentation is isolated to a specific sleep stage. The third type of partial SD is named sleep restriction or sleep debt, which is characterized by reduced sleep duration [3]. The first and the last types are the most prevalent conditions.

Thus, sleep deprivation or restriction is related to the total sleep time, loss of one or more sleep phases and also with the sleep quality. Therefore, low sleep quality is a type of sleep deprivation. Research has shown that inadequate sleep can affect vigilance, information integration, reasoning abilities and motor control performance, such as posture control. To maintain effective performance, sleep quality is as important as sleep quantity. Consequently, subjects should be assessed about their sleep patterns such as sleep interruptions, sleep latency, total sleep time, and frequency of awakenings.

However, the objective assessment of sleep quality is not trivial and it has not received the same attention as acute SD. In fact, to our knowledge, there is no research about the effect of chronic SD and the reduction of sleep quality on PC. In this respect, we hypothesize that chronic partial sleep deprivation and the reduction of sleep quality can affect negatively postural control. Therefore, the objective of the present study is to assess experimentally the effects of the decrease of quality of sleep on PC.

## Material and Methods

### Subjects

Thirty healthy volunteers of both genders participated in the study (18–29 years; 24 women and 6 men). The exclusion criteria were medical history of musculoskeletal, rheumatological, neurological, visual, vestibular, psychiatric diseases and/or with diabetes mellitus, a body mass index (BMI) smaller than 18.5 or larger than ≥ 30 (obesity) kg/m^2^. This study was approved by the ethics committee of Federal Institute of Education, Science, and Technology of Southeast of Minas Gerais, and all subjects gave their written informed consent.

#### Physical Activity Level

The level of physical activity was assessed using the International Physical Activity questionnaire (IPAQ) [25], adapted to Brazilian Portuguese version [26]. This questionnaire estimates the weekly time spent sitting and in moderate and vigorous activities, in different contexts (occupation, transportation, household activities or leisure). The metabolic equivalent of task (MET) is calculated by multiplying the activity duration, intensity (weekly), and frequency (moderate and vigorous walking). The weekly MET can be estimated by adding the values spent in each activity.

### Sleep and circadian rhythm assessment protocol

#### Actigraphy

The sleep and the circadian rhythm of the participants were measured using a wrist actigraph ActTrust (Condor Instruments®) for more than a week [27, 28]. The actigraph was worn continuously and it was removed only when the activities involved water immersion. Actigraphy data were collected for each 1-minute epoch and analyzed in the ActStudio (Condor Instruments®) software. The sleep and circadian rhythm parameters were based on the movement and temperature of the subject along with the ambient light data recorded by the actigraph following standard procedures in sleep actigraphy [27, 28]. The Proportional Integration Mode (PIM) algorithm was used to process the acceleration to obtain a measure of user activity. The PIM data with epochs of 60 s were integrated every hour to generate 24 epochs of 3600 s each day. The sleep and circadian rhythm parameters were [27, 28]:

- Total sleep time (TST): the time, in minutes, of the major interval between the start and end of sleeping each day.
- Wake After Sleep Onset (WASO): number of minutes awake (as detected by the actigraph) within the major interval defined by the TST.
- Sleep efficiency: ratio of TST to total bed time. The total bed time is the interval, in minutes, between the instant that the subject lies on the bed until the moment he/she stands after a major sleep interval.
- L5m: It is the sum of the activity of the least active 5-h period across a day. This variable is calculated daily and used the average of all day resulting in L5m.

### Questionnaires

#### Morningness–eveningness questionnaire (HO)

The morningness–eveningness questionnaire (HO) from Horne and Östberg [29] was used in a Brazilian version [30] to assess if the circadian rhythm of the participant had a peak alertness in the morning, in the evening, or in between. The subject is asked to indicate when, he/she would prefer to wake up or start sleep, according to time availability. The score is added together and the sum converted into a five-point morningness–eveningness scale: a) 16 to 30 score “definitely evening type”; b) 31 to 41 score “moderately evening type”, c) 42 to 58 score “neither type; d) “59 to 69 score “moderately morning type; and e) 70 to 86 score “definitely morning type”.

#### Pittsburgh Sleep Quality Index

The Pittsburgh Sleep Quality Index (PSQI) is a self-rated questionnaire which assesses sleep quality and disturbances over a 1-month time interval [31, 32]. Nineteen individual items generate seven scores about sleep: subjective sleep quality, sleep latency, sleep duration, usual sleep efficiency, sleep disturbances, use of sleeping medication, and daytime dysfunction. The sum of scores for these seven components yields one global score. Scoring of the answers is based on a 0 to 3 scale and a global sum equal or larger than 5 indicates a “poor” sleeper.

#### Epworth Sleepiness Scale (ESS)

The ESS [32, 33] is a self-administered questionnaire that measures the general level of daytime sleepiness of the participants that have to rate, on a 4-point scale (0–3), their usual chances of dozing off or falling asleep. A higher score indicates higher level of daytime sleepiness. Using a cut-off score >10, it is possible to identify individuals with excessive daytime sleepiness. Scores >16 indicate severe sleepiness.

### Posture measuring protocol

#### Equipment

The Biodex Balance System® (BBS, Biodex Inc Shirley, NY USA) is a device to measure postural sway as well as train and assess balance [5, 34]. It contains four strain gauges under a circular platform to measure the displacement of center of pressure (COP) at a sampling rate of 20 Hz. Eight programmable springs located underneath the outer edge of the platform allows it to tilt with twelve levels of resistance or to be locked for static measurements [34]. The platform stability levels range from 1 (lowest rigidity) to 12 (highest rigidity) and static.

The BSS device is interfaced with a dedicated software (Biodex, version 1.08, Biodex Inc, Shirley, NY USA). It measures the platform tilt in each axis and provides a postural sway score based on the variance of the platform displacement, the stability index (SI). There is a stability index for the sagittal plane (antero-posterior, APSI) for the frontal plane (medio-lateral, MLSI) and global one (overall, OSI). Higher values of SI indicate worse postural control as this displacement is related to the COP excursions. Another outcome variable is the sway index that is the standard deviation of the stability index and it is provided in the static platform tests.

#### Testing procedures

**Fig 1:**
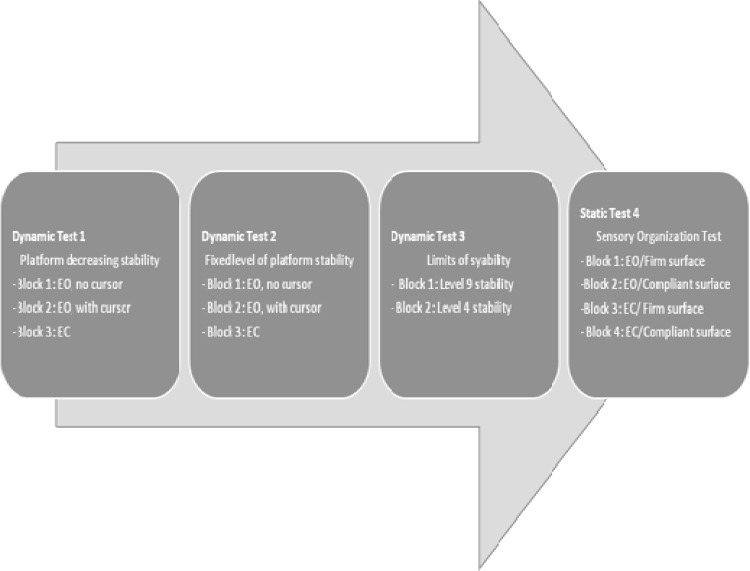
Sequence of postural control tests. EO: eyes open; EC: eyes closed.

The subjects were asked to stand barefoot on the platform with the knees slightly flexed, in a relaxed position with arms the hanging. For safety reasons, the volunteers were allowed to touch the support rails to restore balance during extreme postural deviations and this event was noted as a balance loss. Participants were instructed to adjust their preferred feet position with the platform allowed to tilt, in such a way that they could maintain stability. A grid printed on the platform was used to code this feet position that had to be maintained until the completion of the test. A familiarization trial was conducted in which the participants were introduced to the four testing protocols, that were recorded during a single session on the same day. It took less than 2 hours to complete the tests, including filling the questionnaires. All the subjects performed the sequence of tests in the same order (Fig 1).

The four testing protocols assess the influence of different factors on human postural control, such as sensory inputs, but also the ability to react to changes in the base of support (platform tilt) and to actively control posture (control a cursor on the screen with the position of the COP). To do so, the participant is required to maintain standing balance under conditions that alter sensory inputs, e.g. eyes closed require the integration of vestibular and somatosensory inputs. In the cursor condition, the subject has to control a cursor displayed on the screen with the position of the COP, in this case the ability to actively control posture is assessed and it requires attention and concentration. These conditions form the dynamic tests 1 and 2. In the dynamic test 3, the limits of stability control are tested in a serious game. This game motivates the subject and increases alertness, attention and physical performance. The static test is a classic sensory integration test for PC that is based on the manipulation of sensory inputs with eyes open and close and rigid or soft foam under the foot to remove plantar pressure sensation while keeping the platform fixed or static.

Dynamic Test 1- Decreasing platform stability levels: The platform was set to go from level 12 (highest stability—the most rigid condition) to level 1 (lowest stability) in 30 seconds. The participants completed 3 blocks of tests with 5 repetitions/each, as follows: block 1 with eyes open without cursor; block 2 with eyes open with cursor; and block 3 with eyes closed.

In the first block, with eyes open, the volunteers concentrated on the screen without any visual feedback; in the second block, the participants were instructed to position a cursor displayed on the screen, that represents the COP, near the center of the target. In block 3, the participants kept their eyes closed for 30s. The overall, the anterior-posterior and the medio-lateral stability scores were calculated as the mean of the five repetitions. After the third block the participants rested sitting for 2 minutes.

Dynamic test 2- Constant platform stability level: the platform was set to remain in the same level of stability for 30 s. The order of stability levels was 1, 2, 3, 4 and 9, for each of the three blocks (1-eyes open; 2-cursor; 3-eyes closed). The volunteers performed one repetition at each level. The overall, anterior-posterior and medio-lateral stability rates were obtained by the average of the 5 levels. The number of balance loss occurrences (touching or holding the support rail of the equipment) and the duration of time standing on the platform before the first loss of balance episode were also recorded. The participants rested sitting for 2 minutes before the following test.

Dynamic test 3- Constant platform stability level: during each of the four trials, two with the platform stability level 9 and two in level 4, the volunteers had to control their COP. They had to move as fast as possible a cursor that represented their own COP from a central starting point to one of eight targets, equally spaced on a circle, which flashed in a random order. When the cursor was in the target for 0.25 s it stopped flashing. A movement precision index percentage was generated considering 100% the straight line between the center and the target, both overall (global) and for each direction (forward, backward, right, left, forward/right, forward/left, backward/right, backward/left). The means of the two trials of levels 9 and 4 for the movement precision index in each direction were retained for further analysis. After these tests the participants rested sitting for 2 minutes.

Static Test–Sensory integration test: the protocol consists of four conditions under a static surface and provides information on the balance of the individuals involving visual, proprioceptive and vestibular control. The conditions are as follows: eyes open firm surface, eyes closed firm surface, eyes open foam surface, eyes closed foam surface. The Biodex software generates a postural sway index for each condition. In the present study, four conditions were performed twice, following the order described above. The postural sway index obtained, that is calculated as the standard deviation of the stability index, was the mean of the two repetitions.

### Data analyses

The parameters obtained from the questionnaires, actigraphy and posture assessment were included in the database for statistical analyses with SPSS package (SPSS Inc. Released 2008. SPSS Statistics for Windows, Version 17.0; Chicago: SPSS Inc).

The first step was to classify the people in groups according to their sleep quality (TST, WASO, and efficiency) and circadian patterns (L5) obtained from actigraphy.

The Pearson r correlation was used to determine the strength of the association between in the sleep quality and the rhythm variables. The highest significant correlations were found between L5 and WASO (r = 0.60) e L5 and efficiency (r = 0.65). Therefore, a measure of the circadian pattern L5, could be considered as a collective variable that measures the circadian rhythm and sleep quality. Taking this into account, the subjects were clustered in two groups according to L5, Group 1 with low L5 indicating higher sleep quality and Group 2 with a high L5 and lower sleep quality.

Afterwards, an analysis of Variance (ANOVA) was used to compare between the two groups the parameters that complied with the ANOVA assumptions such as BMI, age, sleep, circadian and PC variables. The data from questionnaires about sleep condition (HO, PSQI, EES, IPAQ/METs) were analyzed by means of nonparametric tests (Kolmogorov-Smirnov Z).

## Results

There were no significant differences between both groups for the parameters evaluating the characteristics of the participants (gender, age, height, weight, days with actigraphy, morning/evening chronotype by HO questionnaire; and level of physical activity by IPAQ METs) except BMI, that was significantly larger for Group 2. Nevertheless, both means were within the normal healthy weight below 25 Kg/m^2^ (Table 1). There were also no differences in the global sleep quality by PSQI (Table 1).

**Table 1:** Physical and sleep characteristics of the participants.

	Group 1 (n = 19)		Group 2 (n = 11)		
	L5 low		L5 high		
**Individual characteristics of sample**					
	**Mean**	**SD**	**Mean**	**SD**	**ANOVA**
Age (years)	21	2.57	22	3.97	
Gender (M)^†^	21.05%		18.18%		
Height (meter)	1.62	0.09	1.65	0.06	
Weight (Kilogram)	58.6	6.85	66.79	7.64	
BMI (Kg/m^2^)	22.22	1.73	24.56	2.18	0.003*
Days with actigraphy	11.21	2.9	12.27	3.23	0.36
**Questionnaires**					
	**Median**	**Min-Max**	**Median**	**Min-Max**	**Mann-Whitney**
HO questionnaire	50	32–70	55	32–69	0.49
PSQI	6	3–19	6	2–11	0.67
ESS	9	4–16	10	6–20	0.10
METs/weekly	1927.5	363–5272.5	2088	612–11305	0.50
**Sleep parameters**					
	**Mean**	**SD**	**Mean**	**SD**	**ANOVA**
TST (min)	494.26	46.39	495.96	58.98	0.93
WASO	7.02	3.22	14.56	9.19	0.003*
Sleep efficiency	0.99	0.005	0.97	0.159	0.001*
**Circadian rhythm parameters**					
	**Mean**	**SD**	**Mean**	**SD**	**ANOVA**
L5	29303.72	7259.64	66647.8	11318	<0.0001*

L5: least active 5-h period; M: male; HO: Horn and Östberg; PSQI: Pittsburgh sleep quality index; ESS: Epworth sleepiness scale; MET: metabolic equivalent; TST: total sleep time; WASO: wake after sleep onset

† data described in relative frequency; min: minutes; SD: standard deviation

* statistically significant difference.

Sleep fragmentation and quality variables, WASO and sleep efficiency were, respectively, higher and lower in Group 2, indicating that they had worse sleep conditions than Group 1 which, in addition, had lower L5 values that measures of sleep restriction and sleep quality (Table 1).

In the dynamic test 1 for PC, Group 2 presented worse performance than Group 1, except for the tests with the cursor (Table 2).

**Table 2:** Stability index score with decreasing platform stability (30 s tests from level 12 to 1. Dynamic test 1).

	Group 1 (n = 19)		Group 2 (n = 11)		p-value (ANOVA)
	L5 low		L5 high		
	Mean	SD	Mean	SD	
	**Eyes open**				
OSI	1.43	0.35	1.68	0.61	0.02*
APSI	1.05	0.32	1.19	0.54	0.37
MLSI	0,77	0.31	1.16	0.49	0.01*
	**Cursor**				
OSI	0.76	0.2	0.88	0.29	0.19
APSI	0.52	0.15	0.61	0.26	0.24
MLSI	0.44	0.15	0.5	0.12	0.32
	**Eyes closed**				
OSI	3.62	1.14	5	0.84	0.002*
APSI	2.49	0.82	3.52	0.78	0.001*
MLSI	2.16	0.7	2.78	0.58	0.02*

L5: least active 5-h period; OSI: overall stability index; APSI: anteroposterior stability index; MLSI: mediolateral stability index; SD: standard deviation

*statistically significance difference.

In the dynamic test 2, there were significant differences in all the stability index scores for the tests with eyes closed (Table 3). The table also includes the number of balance losses. Data from three subjects of Group 1 and one subject of Group 2 were discarded due to errors in this dynamic test.

**Table 3:** Stability index score with stable platform levels (one 30 s test in level 1, 2, 3, 4, and 9. Dynamic test 2).

	Group 1 (n = 16)		Group 2 (n = 10)		p-value (ANOVA)
	L5 low		L5 high		
	Mean	SD	Mean	SD	
	**Eyes open**				
OSI	1.48	0.48	1.87	0.58	0.08
APSI	1.12	0.45	1.48	0.5	0.07
MLSI	0.73	0.26	0.84	0.31	0.36
Time until first balance loss (sec)	30	0	29.5	1.7	0.21
Number of balance losses / Number of trials	0/80		7/50		
	**Cursor**				
OSI	0.83	0.3	0.99	0.36	0.23
APSI	0.59	0.23	0.66	0.23	0.45
MLSI	0.47	0.15	0.6	0.22	0.09
Time until first balance loss (sec)	30	0	29.56	1.39	0.15
Number of balance losses / Number of trials	0/80		1/50		
	**Eyes closed**				
OSI	5.45	1.84	7.41	1.6	0.01*
APSI	3.54	1.25	4.9	0.99	0.008*
MLSI	3.34	1.16	4.5	1.2	0.02*
Time until first balance loss (sec)	19.77	6.1	20.4	7.79	0.82
Number of balance losses / Number of trials	215/80		116/50		

L5: least active 5-h period; OSI: overall stability index; APSI: anteroposterior stability index; MLSI: mediolateral stability index; sec: seconds; SD: Standard deviation

*statistically significance difference.

In the dynamic test 3—stability limits—there was an only significant difference between the groups in overall precision index for level 4 of stability (p = 0.03). In the static test 4- sensory integration—the only significant difference was found between Groups 1 and 2, with eyes closed on a compliant (foam) surface (Table 4).

**Table 4:** Sway index of Sensory Organization Test (Static test).

	Group 1 (n = 18)		Group 2 (n = 11)		p-value (ANOVA)
	L5 low		L5 high		
	Mean	SD	Mean	SD	
EOFS	0.71	0.25	0.75	0.19	0.66
ECFS	0.89	0.29	0.99	0.23	0.33
EOCS	0.97	0.35	0.97	0.21	0.95
ECCS	2.38	0.72	2.96	0.74	0.04*

L5: least active 5-h period; EOFS: eyes open on firm surface; ECFS: eyes closed on firm surface; EOCS: eyes open on compliant surface; ECCS: eyes closed on compliant surface; SD: standard deviation

* statistically significance difference.

## Discussion

The results show that the group with chronic lower sleep quality had a worse performance in the control of posture, in a similar way as subjects that spent one night without sleep. This finding means that subtle (and often neglected) loss of sleep quality results in a loss of performance of an important motor task such as posture control with eyes closed. Although it was proven that PC was affected by one day of complete SD [8–23, 35, 36], this is the first study showing posture deterioration that could be attributed to a chronic decrease in the sleep quality monitored for more than one week.

There are important consequences of these results in the research fields of sleep and motor control. With respect to sleep research it was proven that actigraphy can be used to assess sleep quality. The two parameters obtained by this method, L5 and WASO were useful to detect low sleep quality that affected posture control. It seems relevant to conduct further research to analyze the sensitivity of these parameters.

With respect to the motor control research the evidence provided here supports that a chronic low sleep quality affects the control of posture with eyes closed in healthy young individuals. Therefore, it is crucial to monitor the sleep quality of populations in risk of falling, such as the elderly. Moreover, an important consequence in research and clinical evaluation of posture is that the sleep quality of the subjects should be monitored before the tests.

This study combined PC assessment, sleep questionnaires and objective sleep parameters from 30 participants using a wrist actigraph for more than one week. This procedure provides data about SD and PC that are more realistic than those obtained from to 1 night of acute SD. In this study there were no significant differences in the total sleep time between groups, however, there was a difference in the sleep quality assessed by actigraphy with the L5 variable. This parameter measures the movements during the resting period and is an index of the sleep quality.

Actigraphy is less complex, invasive and expensive than polysomnography (PSG) and multiple sleep latency test (MSLT), that are the gold standards for sleep assessment. More interestingly, actigraphy allows the quantification of important parameters of sleep and wake over long periods without interfering in the daily life of the subjects [21]. The disadvantages are related to the assumption that low activity in the dark means sleep as well as the lack distinction between sleep stages, respiratory disorders or abnormal movements [37].

In addition to objective assessment, questionnaires were used in this study to evaluate subjective sleep quality was evaluated in this study using the Pittsburgh Sleep Quality Index (PSQI); the general level of daytime sleepiness was assessed with the Epworth Sleepiness Scale (ESS). The PSQI and the Pittsburgh Sleep Diary (PghSD) are instruments used to screen sleep problems [20, 22]). The ESS is used in studies on acute SD to evaluate the subjective perception of sleepiness [14]. Alertness is the opposite of sleepiness and is one of the factors directly related to PC performance [38].

In the present study, different factors related to the control of posture have been manipulated. The sensory inputs, vision (eyes open or closed) as well as footsole proprioception (firm or compliant surface) were combined and there were also different stability levels in the platform (Biodex Balance System®). These combinations of platform stability and sensory inputs set different levels of complexity for the PC tests from the easiest one on a firm static platform with visual feedback (static test) to the more difficult ones with the eyes closed and the platform can tilt (dynamic test 2).

There were blocks on the dynamic tests (block 2 in tests 1 and 2) that required to move a cursor on the screen by displacing the COP. These tests demanded attention and provided motivation to the participants. This possibly increased their alertness level and masked any effect of sleep quality on the PC tests.

The more difficult tests that had different platform stability levels (dynamic test 2) showed significant differences between groups with eyes closed but not with eyes open, and similar results were reported for PC tests after one night SD [17–23]. This suggests that lack of vision impairs postural balance more deeply in subjects with chronic problems of sleep quality, maybe due to disturbances in the brain sensory integration areas. Analysis of brain activity has shown that the areas of the cerebral cortex that regulate aspects of attention, alertness and cognitive ability, such as thalamus and regions within the pre-frontal cortex, undergo deactivation periods following 24 h of sleep deprivation [39] and decrease activity further as the period of SD increases [40].

Another aspect to mention is that the classification on different sleep quality groups have been based on the parameter L5. It measures the movement activity intensity during the least active 5h of the day, so it is also related to the circadian rhythm. It reveals a sleep with increased motor activity that is associated with poor sleep quality [41]. It is also related to other sleep parameters, such as fragmentation (WASO) and sleep efficiency, that were also significantly different between these groups. Group 2, with large values of L5, higher fragmentation (WASO) and lower efficiency, showed worse performance than Group 1 (higher efficiency with lower L5 and WASO).

An increase of sleep movements and fragmentation might force the person to restart the sleep cycle and decrease the time spent in the REM (Rapid Eye Movement) sleep phase. It seems that an adequate REM sleep phase is important for motor control because it has an impact on the proper regulation of muscle tonus. This effect could be regarded as a new function of REM sleep and could explain the better performance of Group 1 comparing to Group 2 [42]. It must be noted that in the present study, several confounding variables in PC and sleep such as gender [5], age [20], chronotype [43], level of physical training [44], and BMI [45, 46] were measured and controlled. While age and gender were usually reported in all the studies, the other three are rarely or never addressed and it is suggested to include them in future research. Although there were differences in absolute values of BMI between groups 1 and 2 all the subjects were within the ‘eutrophy’ category.

The present study has some limitations. The direct comparison of the COP variables in this study with those of others is not straightforward due to the difference between the Biodex Balance System® (BBS) and the conventional force platforms. The BBS is an instrument for clinical applications and it is comparable to other balance measures currently in use [47].

Another limitation of the study was the different sample size between the groups. This is due to the unknown sleep quality parameters when recruiting the volunteers. Only after monitoring the sleep for several days it was possible to determine if the subject belonged to the low sleep quality group or not. The differences in same sizes may reflect the differences in sleep quality of a general population of young healthy adults, so it could not be solved by increasing the sample size. Actually, with a larger sample it could be possible to draw more reliable conclusions about the sleep quality of the population group.

This is a pioneer study in which individuals without subjective sleep complaints were monitored for more than one week and performed a series of PC tests. As a result, participants with sleep inefficiency, characterized by fragmentation and excessive nighttime movements, had PC performance similar to subjects that suffered one night of artificial total SD. Therefore, we can conclude that the effects of decreased sleep efficiency on posture control are relevant and cannot be neglected.

The motor performance disorders caused by chronic low sleep quality could be critical in the case of athletes or military personnel. In this respect, it must be noted that, in this study, both groups had approximately the same sleep hours (TST) and that they had no significant differences in the questionnaires that assessed the sleep quality (PSQI) and the sleepiness (ESS). Thus, the lack of sleep quality was unnoticed by the participants.

Further research in this area should include individuals with chronic partial SD considering age and BMI along with the execution of dual-task activities /tests under different sensory information conditions.

## Supporting Information

S1 Table Statistical description of the data (Mean, variance, maximum and minimum). BMI: body mass index; TST: total sleep time; WASO: wake after sleep onset; L5: least active 5-h period; OSI: overall stability index; APSI: anteroposterior stability index; MLSI: mediolateral stability index; EOFS: eyes open on firm surface; ECFS: eyes closed on firm surface; EOCS: eyes open on compliant surface; ECCS: eyes closed on compliant surface.
(DOCX)
