# Supplementary material for: Chronic Low Quality Sleep Impairs Postural Control in Healthy Adults
Source: PLoS One. 2016 Oct 12;11(10):e0163310. doi: 10.1371/journal.pone.0163310 (PMC5061348; doi:10.1371/journal.pone.0163310)
Supplement: S1 Table — BMI: body mass index; TST: total sleep time; WASO: wake after sleep onset; L5: least active 5-h period; OSI: overall stability index; APSI: anteroposterior stability index; MLSI: mediolateral stability index; EOFS: eyes open on firm surface; ECFS: eyes closed on firm surface; EOCS: eyes open on compliant surface; ECCS: eyes closed on compliant surface. (DOCX) [file pone.0163310.s001.docx]

|  |  | **Group 1 (n=19)** | | | | **Group 2 (n=11)** | | | |
| --- | --- | --- | --- | --- | --- | --- | --- | --- | --- |
|  |  | Median | Variance | Minimum | Maximum | Median | Variance | Minimum | Maximum |
|  |  |  |  |  |  |  |  |  |  |
|  | Age | 21.00 | 6.62 | 18.00 | 29.00 | 20.00 | 15.80 | 19.00 | 29.00 |
|  | Height | 1.61 | 0.01 | 1.48 | 1.80 | 1.65 | 0.00 | 1.52 | 1.75 |
|  | Weight | 56.45 | 46.88 | 48.40 | 74.50 | 64.30 | 58.33 | 58.00 | 84.00 |
|  | BMI | 21.97 | 3.01 | 19.20 | 26.60 | 25.10 | 4.75 | 20.37 | 27.45 |
|  | TST | 490.83 | 2152.24 | 382.59 | 585.57 | 501.45 | 3478.32 | 390.38 | 598.36 |
|  | WASO | 7.25 | 10.37 | 2.73 | 15.20 | 12.18 | 84.48 | 3.25 | 36.79 |
|  | Sleep Efficiency | 0.99 | 0.00 | 0.97 | 0.99 | 0.97 | 0.00 | 0.94 | 0.99 |
|  | L5 | 28906.58 | 52702362.11 | 13403.82 | 42082.63 | 65124.39 | 128097349.52 | 53823.67 | 88724.87 |
| **Dynamic Test #1** | Eyes open OSI | 1.38 | 0.12 | 0.88 | 2.10 | 1.60 | 0.37 | 1.12 | 2.94 |
|  | Eyes open APSI | 0.96 | 0.10 | 0.64 | 1.74 | 1.02 | 0.29 | 0.48 | 2.42 |
|  | Eyes open MLSI | 0.76 | 0.09 | 0.34 | 1.54 | 0.92 | 0.24 | 0.68 | 2.08 |
|  | Cursor OSI | 0.74 | 0.04 | 0.48 | 1.26 | 0.76 | 0.09 | 0.54 | 1.62 |
|  | Cursor APSI | 0.50 | 0.02 | 0.32 | 0.82 | 0.56 | 0.07 | 0.28 | 1.30 |
|  | Cursor MLSI | 0.44 | 0.02 | 0.26 | 0.82 | 0.46 | 0.01 | 0.34 | 0.72 |
|  | Eyes closed OSI | 3.40 | 1.30 | 1.46 | 6.68 | 5.04 | 0.88 | 3.48 | 6.30 |
|  | Eyes closed APSI | 2.26 | 0.67 | 0.88 | 4.52 | 3.62 | 0.60 | 2.44 | 4.92 |
|  | Eyes closed MLSI | 1.98 | 0.50 | 0.96 | 3.78 | 2.78 | 0.34 | 2.00 | 3.78 |
| **Dynamic Test #2** | Eyes open OSI | 1.33 | 0.23 | 0.84 | 2.50 | 1.72 | 0.34 | 1.16 | 3.20 |
|  | Eyes open APSI | 1.01 | 0.21 | 0.58 | 2.06 | 1.42 | 0.25 | 0.90 | 2.66 |
|  | Eyes open MLSI | 0.66 | 0.07 | 0.36 | 1.18 | 0.78 | 0.10 | 0.50 | 1.34 |
|  | Eyes open time balance loss | 30.00 | 0.00 | 30.00 | 30.00 | 30.00 | 2.92 | 24.60 | 30.00 |
|  | Cursor OSI | 0.80 | 0.09 | 0.40 | 1.62 | 0.85 | 0.13 | 0.56 | 1.62 |
|  | Cursor APSI | 0.55 | 0.05 | 0.30 | 1.22 | 0.58 | 0.05 | 0.40 | 1.04 |
|  | Cursor MLSI | 0.45 | 0.02 | 0.20 | 0.82 | 0.51 | 0.05 | 0.36 | 1.00 |
|  | Cursor time balance loss | 30.00 | 1.00 | 30.00 | 34.00 | 30.00 | 1.94 | 25.60 | 30.00 |
|  | Eyes closed OSI | 6.15 | 3.39 | 2.54 | 8.46 | 7.41 | 2.59 | 5.08 | 9.78 |
|  | Eyes closed APSI | 3.58 | 1.57 | 1.64 | 5.64 | 5.04 | 0.97 | 3.30 | 6.18 |
|  | Eyes closed MLSI | 3.50 | 1.35 | 1.64 | 5.66 | 4.29 | 1.45 | 3.06 | 6.28 |
|  | Cursor time balance loss | 19.90 | 37.22 | 8.60 | 28.20 | 21.20 | 60.67 | 10.20 | 30.00 |
| **Static Test** | EOFS | 0.68 | 0.06 | 0.30 | 1.38 | 0.74 | 0.04 | 0.49 | 1.13 |
|  | ECFS | 0.84 | 0.08 | 0.46 | 1.53 | 1.06 | 0.05 | 0.67 | 1.32 |
|  | EOCS | 0.90 | 0.12 | 0.51 | 1.82 | 0.94 | 0.05 | 0.60 | 1.28 |
|  | ECCS | 2.34 | 0.52 | 1.26 | 4.00 | 2.98 | 0.55 | 2.00 | 4.77 |

BMI: body mass index; TST: total sleep time; WASO: wake after sleep onset; L5: least active 5-h period; OSI: overall stability index; APSI: anteroposterior stability index; MLSI: mediolateral stability index; EOFS: eyes open on firm surface; ECFS: eyes closed on firm surface; EOCS: eyes open on compliant surface; ECCS: eyes closed on compliant surface
